# Activity of the novel polo-like kinase 4 inhibitor CFI-400945 in pancreatic cancer patient-derived xenografts

**DOI:** 10.18632/oncotarget.13619

**Published:** 2016-11-25

**Authors:** Ines Lohse, Jacqueline Mason, Pinjiang Cao Mary, Melania Pintilie, Mark Bray, David W Hedley

**Affiliations:** ^1^ Ontario Cancer Institute and Campbell Family Cancer Research Institute, Princess Margaret Cancer Centre, University Health Network, Toronto, Ontario, Canada; ^2^ Department of Medical Oncology and Haematology, Princess Margaret Cancer Centre, Toronto, Ontario, Canada

**Keywords:** polo-like kinase 4, PLK4, CFI-400945, pancreatic cancer, patient-derived pancreatic cancer xenografts

## Abstract

**Background:**

Polo-like kinase 4 PLK4 plays a key role in centriole replication. Hence PLK4 inhibition disrupts mitosis, and offers a novel approach to treating chromosomally unstable cancers, including pancreatic cancer. CFI-400945 is a first in class small molecule PLK4 inhibitor, currently undergoing early phase clinical trials.

**Results:**

Treatment with CFI-400945 significantly reduced tumor growth and increased survival in four out of the six models tested. Consistent with PLK4 inhibition, we observed reduced expression of the proliferation marker Ki-67 associated with an increase in nuclear diameter during treatment with CFI-400945. Additionally, treatment with CFI-400945 resulted in a significant reduction of tumor-initiating cells.

**Discussion:**

These results support the further investigation of PLK4 as a drug target in pancreatic cancer.

**Methods:**

Sensitivity to CFI-400945 was tested in a series of six patient-derived pancreatic cancer xenografts, selected to represent the range of growth characteristics, genetic features, and hypoxia found in pancreatic cancer patients.

## INTRODUCTION

The genomic landscape of pancreatic cancer is dominated by a high prevalence of mutations involving K-Ras, p53, SMAD4, and p16 [1, 2], none of which is readily targetable using currently available agents. An emerging picture from ongoing sequencing studies indicates that these cancers are characterized by an unusually high level of chromosomal instability [1, 2]. It is likely that this accounts, at least in part, for their rapid progression towards the aggressive, drug-resistant phenotype observed in the clinic. Recently it has been suggested that chromosomal instability causes vulnerability to agents that disrupt orderly movement through the late cell cycle, suggesting a novel approach to the treatment of these highly lethal cancers.

Polo-like kinases (PLKs) form a family of structurally related mitotic kinases with diverse functions in the normal regulation of the late cell cycle [3, 4]. All members of the PLK family have been shown to be dysregulated in several types of cancer [5, 6, 7], and they are considered to be candidate drug targets. Polo-like kinase 4 (PLK4) plays a unique role in centriole regulation [8]. Genetic manipulations that deplete PLK4 disrupt centriole duplication, whereas overexpression causes centrosome amplification. Although PLK4 inhibition would be expected to disturb mitosis in normal cells, potentially this would be more severe in chromosomally-unstable cancers, providing a clinically-useful therapeutic window. CFI-400945 is a potent PLK4 inhibitor that is currently undergoing a phase I clinical trial [9, 10, 11]. Treatment with CFI-400945 *in vitro* led to aberrant centriole duplication and abnormal mitoses resulting in cell death or cell-cycle arrest [9]. Preclinical testing showed single agent activity against patient-derived breast cancer xenografts [9]. This study showed an increased sensitivity of PTEN-null tumors to PLK4 inhibition using CFI-400945, suggesting that PTEN deficiency may be a predictive biomarker of CFI-400945 response [9]. In the present study we therefore tested CFI-400945 for activity in a series of orthotopically-grown, patient-derived pancreatic cancer xenografts, selected to represent the range of genetic and growth characteristics seen in the clinic.

## RESULTS

### Characterization of primary patient-derived xenografts

**Figure 1:**
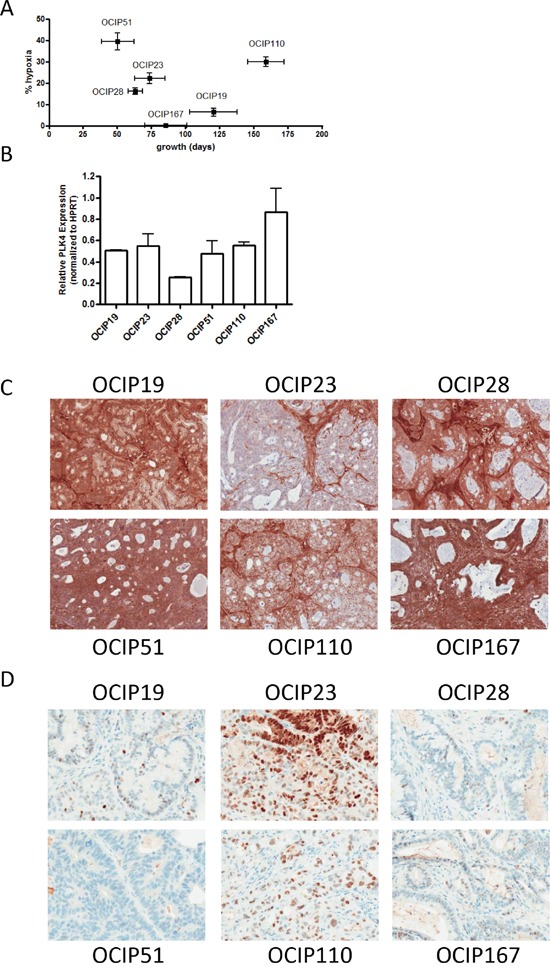
Patient-derived pancreatic xenografts **A.** The PDX models used were chosen to represent a range in growth rate defined as the time elapsed between two passages, and magnitude of hypoxia as evaluated by EF5 staining. **B.** PLK4 mRNA expression was examined using RTPCR. Expression of **C.** PTEN and **D.** p53 was evaluated using immunohistochemistry. Bars represent 100μm.

Tumor growth rate varied from 50 days in OCIP51 to 157 days in the slow growing model OCIP110, and the magnitude of hypoxia from 1% in the least hypoxic model OCIP167 to 40% in OCIP51 (Figure 1; Table 1). These PDX models showed a range of PLK4 expression, with OCIP28 showing the lowest and OCIP167 the highest expression of PLK4 mRNA (Figure 1B). With the exception of OCIP23, they showed expression of PTEN, using tumor-associated stroma as internal positive control, although in OCIP110 PTEN staining was appreciably less when compared to the stroma (Figure 1C, Table 1). Mutations in p53 were found in OCIP23 and OCIP110, resulting in widespread detection of p53 by IHC (Figure 1D; Table 1). As we reported previously, one of these models, OCIP28, is derived from a patient with a germline BRCA2 mutation [12]. All models show mutation in Kras codon 12.

**Table 1:** Characterization of patient-derived pancreatic xenograft models

Model	Diagnosis	Hypoxia (%)		Passage time (days)		p53 exp.$ IHC	PTEN exp. IHC	BRCA2
		mean	SD	mean	SD			
OCIP19	DAC*	6.3	5.77	120.33	17.47	n+	+ +	wt**
OCIP23	DAC	22.16	6.82	73.8	11.03	y#	-	wt
OCIP28	DAC	14.89	7.02	63.25	5.38	n	+ +	mt##
OCIP51	DAC	39.41	11.64	50.33	11.98	n	+ +	wt
OCIP110	DAC	29.88	9.42	158.75	13.38	y	+	wt
OCIP167	DAC	0.27	0.1	85.5	18.31	n	+ +	wt

* DAC: Ductal Adenocarcinoma;

$ exp. IHC: expression immunohistochemistry;

+ n: no;

# y: yes

** wt: wild type;

## mt: mutant

### Effects of PLK4 inhibitor CFI-400945 on tumor growth

**Figure 2:**
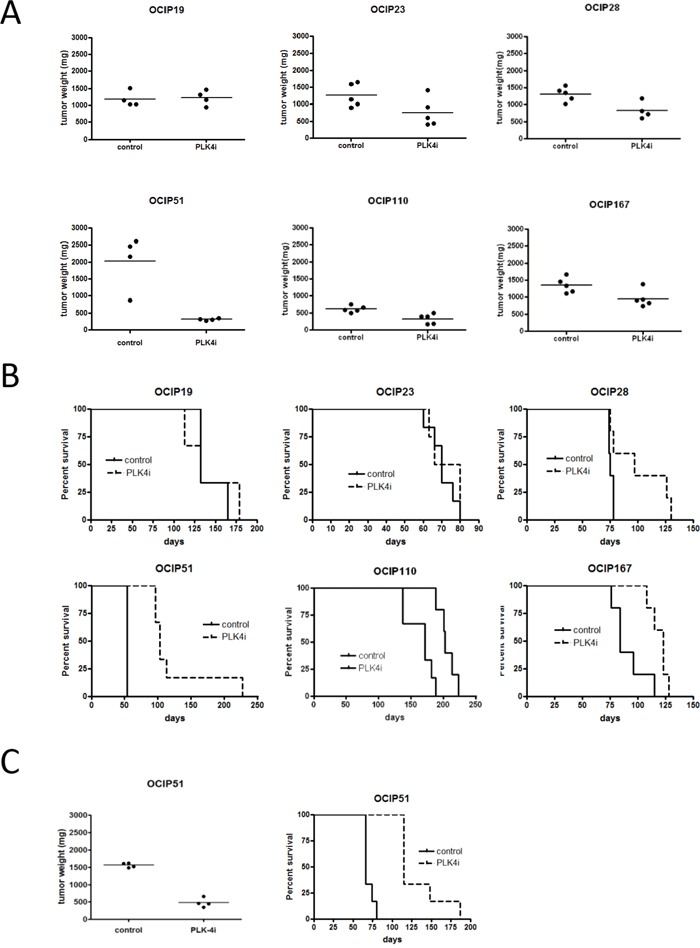
Treatment with CFI-400945 reduces tumor growth Orthotopically implanted animals were treated with either vehicle or CFI-400945 for 21days (n=10 per group). **A.** Tumor weight was evaluated 24h after the last treatment (n=4-5 per group). Treatment significantly reduced tumor growth in OCIP28 (p=0.032), OCIP51 (p=0.029) and OCIP110 (p=0.0079). Tumor volume was also reduced in OCIP167 (p=0.056), although not significantly while no reduction in tumor growth was observed in OCIP19 (p=0.89) and OCIP23 (p=0.095). **B.** The remaining animals were observed until humane endpoint to evaluate survival (n=5-6 per group). Treatment with CFI-400945 had no impact on survival of OCIP19 (p=0.83) and OCIP23 (p=0.53) while survival increased in OCIP28 (p=0.044), OCIP51 (p=0.00091), OCIP110 (p=0.0029) and OCIP167 (p=0.014). **C.** Treatment response and survival was evaluated in animals orthotopically implanted with previously treated OCIP51 tumors (p=0.0004) (n=10 per group).

The six models tested showed a wide range in sensitivity to CFI-400945, as summarized in Figure 2. Tumor weights following three weeks of treatment were significantly reduced (p<0.05) in OCIP28, 51 and 110 compared to vehicle control (Figure 2A). All three of these tumors also showed significant prolongation of survival following treatment with CFI-400945 compared to control, as did a fourth model, OCIP167 that showed borderline significant reduction in tumor weight at three weeks (Figure 2B). Treatment response was not correlated with the expression levels of the drug target PLK4 as assessed by qRT-PCR.

One model, OCIP51, was highly sensitive to CFI-400945, with sustained survival following the initial three weeks of treatment. In order to evaluate whether OCIP51 acquires resistance to CFI-400945, tumors previously treated with one course of CFI-400945 were re-implanted orthotopically. When palpable, animals were treated for a further 21 days with either CFI-400945 or vehicle. Re-treatment with CFI-400945 significantly reduced tumor growth and increased survival when compared to the untreated control (p=0.0004), indicating that the initial exposure to drug did not select for the emergence of drug resistance (Figure 2C). As previously reported [9], treatment with CFI-400945 was well tolerated, with no signs of distress apart from weight loss (Supplementary Figure S1).

### Effects of CFI-400945 on tissue markers of cell growth

**Figure 3:**
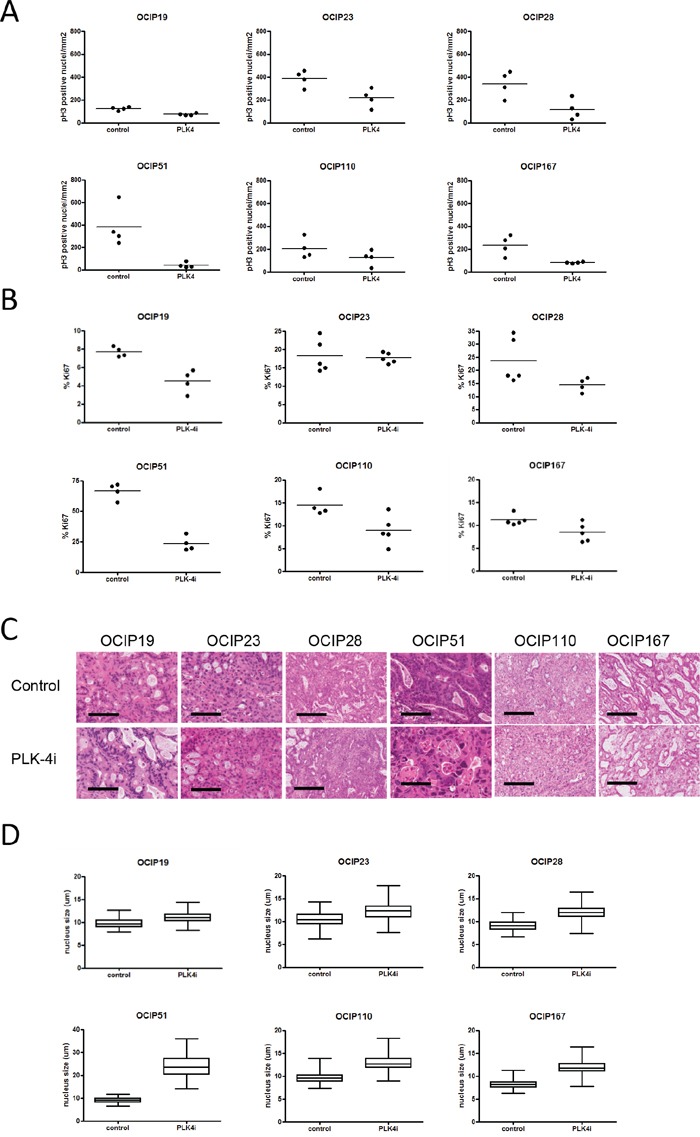
Treatment with CFI-400945 results in reduced tumor cell proliferation Cell proliferation was evaluated using IHC stainings for **A.** pH3 and **B.** Ki67 (n=4-5). pH3 positive cells counted and the total number of pH3 positive nuclei was normalized to the area of viable tumor tissue in order to account for differences in tumor size. Treatment with CFI-400945 reduced the fraction of Ki-67 positive cells in OCIP19 (p=0.029), OCIP28 (p=0.032), OCIP51 (p= 0.029) but not in OCIP23 (p=0.84), OCIP110 (p=0.063), OCIP167 (p=0.095). Phosphorylation of Histon H3 was significantly reduce in OCIP19 (p=0.029), OCIP51 (p=0.029), OCIP167 (p=0.029), but not in OCIP23 (p=0.057), OCIP28 (p=0.057), OCIP110 (p=0.34). **C.** Representative H&E stained sections display the formation of enlarged nuclei in response to treatment with CFI-400945. Bars represent 100μm. **D.** To analyse the effect of CFI-400945 on the size of tumor cell nuclei, the diameter of 100 random tumor cell nuclei per section was measured (n=4-5). Significant increases in nuclei size in response to CFI-400945 were observed in in all models except OCIP19 (OCIP19 p=0.074, OCIP23 p=0.023, OCIP28 p=0.012, OCIP51 p<0.0001, OCIP110 p=0.0052, OCIP167 p<0.0001).

The effects on Ki-67 and pH3 labeling, and nuclear size are summarized in Figure 3. As expected, the baseline levels of the two proliferation markers were roughly concordant with each other, and with the tumor growth rates (Figure 3A and 3B). The effects of treatment with CFI-400945 were variable between the different models. Although broadly consistent with inhibition of proliferation, with the most sensitive model OCIP51 showing the largest decreases in Ki-67 and pH3, significant effects on these markers were also seen in two non-responsive models, OCIP19 and 23.

To investigate whether, in addition to reducing entry into mitosis, treatment with CFI-400945 also influenced progression through mitosis, nuclei positive for pH3 were evaluated for mitotic phases (Supplementary Table S1). Although some tumor showed significant differences in prophase, these changes were model specific and showed no correlation with the response groups established based on tumor growth and survival (Supplementary Table S1, Supplementary Table S2).

While assessing these nuclear markers we noted increases in nuclear size of the treatment groups (Figure 3C). As shown in Figure 3D, the increases in mean nuclear diameter following treatment with the PLK4 inhibitor were statistically significant in all models, with the exception of the least responsive to growth inhibition, OCIP19, and most evident in the highly responsive OCIP51 PDX. In contrast, the levels of cleaved caspase 3, a marker of apoptosis, were unchanged by treatment in all six models (Supplementary Figure S2).

### CFI-400945 treatment reduces tumor-initiating cells

In order to test the effect of CFI-400945 treatment on the tumor-initiating cell (TIC) population, four xenograft models (OCIP19, 23, 51 and 167) were treated with a single dose of 52mg/kg CFI-400945 and processed for limiting dilution assays. Consistent with the growth inhibition results, CFI-400945 treatment of OCIP19 tumors had little effect on the number of TIC. In OCIP23, 51 and 167, on the other hand, TIC frequency was reduced by an order of magnitude in response to treatment with CFI-400945 (Table 2).

**Table 2:** PLK4 inhibitions reduces TIC numbers

Model		TIC number	upper frequency	lower frequency
OCIP19	control	1:568	1:362	1:892
	PLK4i	1:459	1:190	1:1,109
OCIP23	control	1:38	1:17	1:62
	PLK4i	1:175	1:498	1:62
OCIP51	control	1:15	1:10	1:21
	PLK4i	1:417	1:263	1:661
OCIP167	control	1:681	1:278	1:1,669
	PLK4i	1:4,601	1:1,907	1:11,050

## DISCUSSION

The present study evaluates the efficacy of CFI-400945 in a panel of patient-derived pancreatic cancer xenografts displaying a range of growth rates, magnitudes of tumor hypoxia and PLK4 mRNA expression. Treatment with CFI-400945 significantly reduced tumor growth and increased survival in the majority of the tested xenograft models. In addition to its effect on tumor growth, treatment with CFI-400945 results in a significant reduction in tumor-initiating potential. Taken together, this suggests that CFI-400945 treatment targets both the proliferative tumor cell and the TIC population through inhibition of mitotic entry and failure of cytokinesis in response to ablation of spindle formation.

Given the wide range in the response to CFI-400945 in these models, we were interested to identify candidate biomarkers to predict sensitivity in pancreatic cancer patients. The six models used were selected to represent a range of the common mutations linked to pancreatic cancer, and in their growth patterns and the extent of tumor hypoxia, which we have previously shown to be a major correlate of aggressive growth in these models. Interestingly, the most sensitive PDX, OCIP51, also has the most rapid growth, rapid metastasis and greatest level of hypoxia, suggesting that PLK4 inhibition might be particularly effective against clinically aggressive disease. However, a genetic basis for such a link remains elusive, and further research is ongoing [9].

In the present series, expression of the drug target PLK4 did not correlate with sensitivity to CFI-400945. In an earlier publication, Mason et al. [9] suggested that reduced expression of the tumor suppressor PTEN predicted the sensitivity of breast cancers to CFI-400945. However, this appears not to be the case with pancreatic cancers, since the sensitive models express the protein, whereas the only PTEN deficient PDX, OCIP23, was resistant.

Drug resistance, both intrinsic and acquired, appears to be a major reason for treatment failure in pancreatic cancer patients. There appears to be very little intrinsic resistance to PLK4 inhibition based on the effects on tumor cell proliferation and tumor weight observed in this study. Re-challenge of tumors derived from a xenograft tumor previously treated with a full course of CFI-400945 showed only minimal loss of CFI-400945 efficacy, suggesting that treatment with CFI-400945 only leads to minimal development of acquired drug resistance. CFI-400945 is currently undergoing Phase 1 clinical testing (NCT01954316). Based on the results presented here we suggest that CFI-400945 presents a novel therapeutic option for pancreatic cancer patients.

## MATERIALS AND METHODS

### Establishment and characterization of patient-derived xenografts

Patient-derived xenografts (PDX) were established from pancreatectomy samples superfluous to patient diagnosis or ascites samples using a protocol approved by the University Health Network Research Ethics Board. Informed consent was obtained from all participating patients. PDX models were maintained at the orthotopic site in Severe Combined Immune Deficient (SCID) mice according to institutional guidelines for human and animal research, as previously described [13]. Because tumor size is not readily measured at the orthotopic site, growth rates were defined as the average time between two passages. The levels of hypoxia were determined by administration of the 2-nitroimidazole tracer EF5 4 hours prior to sacrifice, and immunohistochemistry using anti-EF5, as previously described [13–17]. Immunohistochemistry (IHC) was used to determine PTEN and p53 status. Briefly, tissue sections were stained for p53 (VP-P958 (clone DO-7), Vector Labs, Burlington, ON, CA, 1:250) or PTEN (#9559, Cell Signaling, Danvers, MA, USA, 1:100) for 2h. After incubation in biotinylated anti-rabbit IgG (BA-1000, Vector Labs, Burlington, ON, CA, 1:200) followed by HRP labeling reagent (Signet Pathology System Inc, Dedham, MA, USA) for 30min, immunoreactivities were revealed by incubation in Nova Red substrate (Vector Labs, Burlington, ON, Canada) for 5min and counterstained in Mayer's haematoxylin. The expression of PLK4 was determined by RT-PCR, using RNA extracted from tumour tissue with the RNeasy mini kit (Qiagen, Toronto, ON, Canada), and cDNA generated using SuperScript Vilo MasterMix (Life Technologies, Burlington, ON, Canada). Triplicate samples were analyzed using TaqMan array fast plates (Life Technologies, Burlington, ON, Canada), and PLK4 expression normalized against HPRT.

### Drug treatment

For single treatments, animals were treated with daily oral gavage of 7.5mg/kg CFI-400945 or saline vehicle control for 21 days. Treatment started when tumors were palpable at a diameter of ~5mm. Animals were treated for three weeks, when the tumors were excised and weighed 24 hr after the last dose in half of each treatment group (n=4-5), with the remaining animals (n=5-6) maintained until the humane endpoint was reached to evaluate survival.

### Assessment of treatment response

In addition to tumor growth inhibition, the tumors were assessed by IHC, using paraffin sections from the treatment and control groups. For each marker, tissue sections from 4-5 individual tumors were analyzed. Proliferation was assessed by extent of Ki-67 staining; mitotic cells were identified by phosphorylated histone H3 Serine-10 (pH3), and cells undergoing apoptosis by cleaved caspase 3 (CC3). Tissue sections were stained for Ki-67 (Novus-Biologicals, Littleton, CO, USA, 1:1000) for 2h or CC3 (#9661, Cell Signaling, Danvers, MA, USA, 1:500) for 30 minutes. For pH3, sections were incubated (3077, Millipore, Etobicoke, ON, CA, 1:50) at room temperature for 1h. Antibody-labeled sections were then processed for IHC as above. Slides were scanned at 20x resolution, or 40x resolution for pH3, using an Aperio Scanscope XT scanner, and the percentage of positive pixels determined using Aperio Imagescope software. Cells stained for pH3 were counted manually, and classified as prophase, metaphase, anaphase and telophase based on morphology. The total number of pH3 positive nuclei was normalized to the area of viable tumor tissue in order to account for differences in tumor size. To measure effects of treatment on nuclear size, the diameter of 100 randomly-selected tumor cell nuclei was measured from 4-5 tumors per model, using the Aperio software.

### Limiting dilution assays

Limiting dilution assays were performed as described previously [14]. Briefly, animals were treated with a single dose of 52mg/kg and tumors harvested 12h after treatment. Single-cell suspensions were prepared, a sample was stained using anti-mouse CD31-FITC, H2K-FITC and propidium iodide, and the percentage of viable cancer cells counted using a hemocytometer. 3-6 dilutions of human cells were injected into the flanks of 3 NOD/SCID mice per dilution and tumor take rate was observed over the course of 6 months. TIC frequency was calculated using the L-Calc software (Stem Cell Technologies, Vancouver, BC).

### Statistical analysis

The pH3 and Ki-67 labeling, and the weights of the tumours were compared utilizing the Mann-Whitney test. The survival percentages were calculated using the Kaplan-Meier method and the log-rank test was applied to test the difference between treatments. Mixed effect models, with animal as a random effect, were employed to determine if treatment affected the diameter of tumor cell nuclei. In order to stabilize the variance, the log transformation was applied to tumor cell nuclei diameter.

## SUPPLEMENTARY MATERIALS FIGURES AND TABLES


