# Supplementary material for: Activity of the novel polo-like kinase 4 inhibitor CFI-400945 in pancreatic cancer patient-derived xenografts
Source: Oncotarget. 2016 Nov 25;8(2):3064–71. doi: 10.18632/oncotarget.13619 (PMC5356865; doi:10.18632/oncotarget.13619)
Supplement: Supplementary file 1 [file oncotarget-08-3064-s001.pdf]

Activity of the novel polo-like kinase 4 inhibitor CFI-400945 in pancreatic cancer patient-derived xenografts

SUPPLEMENTARY FIGURES AND TABLES

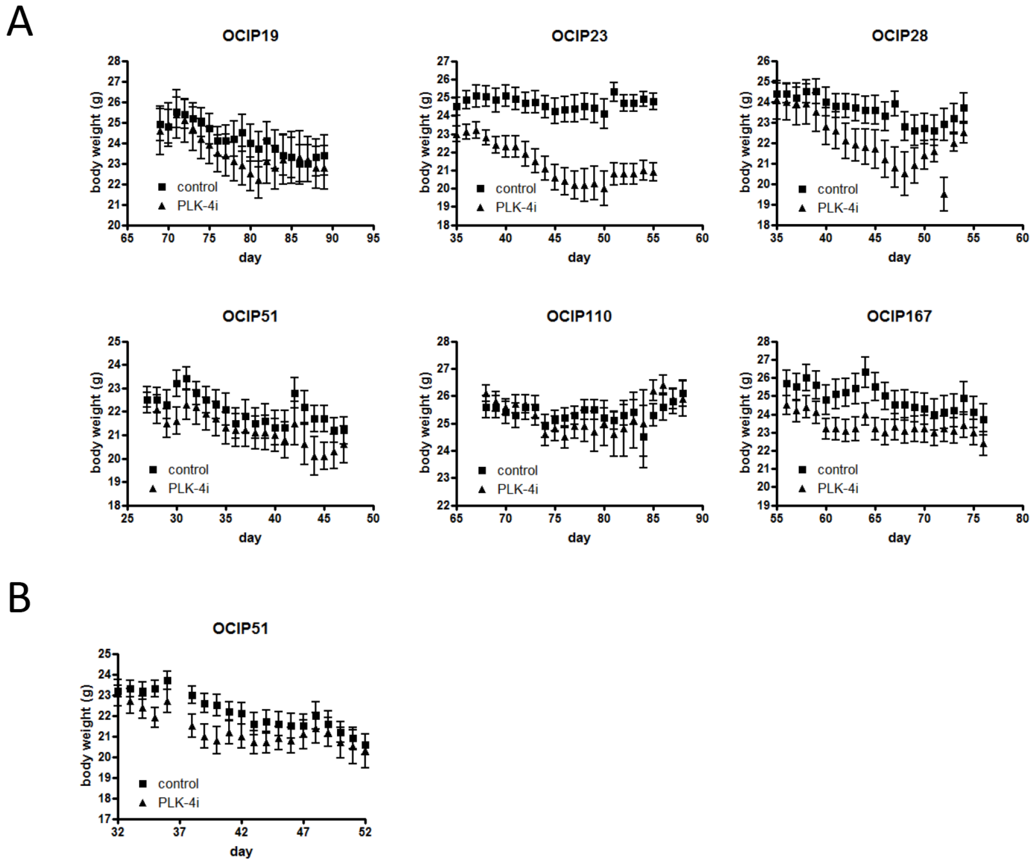

Supplementary Figure S1: Bodyweight of animals treated with CFI-400945. Body weight was monitored over the course of the CFI-400945 treatment in **A.** mice implanted with OCIP19, 23, 28, 51, 110 & 167, **B.** mice implanted with previously treated OCIP51 tumors.

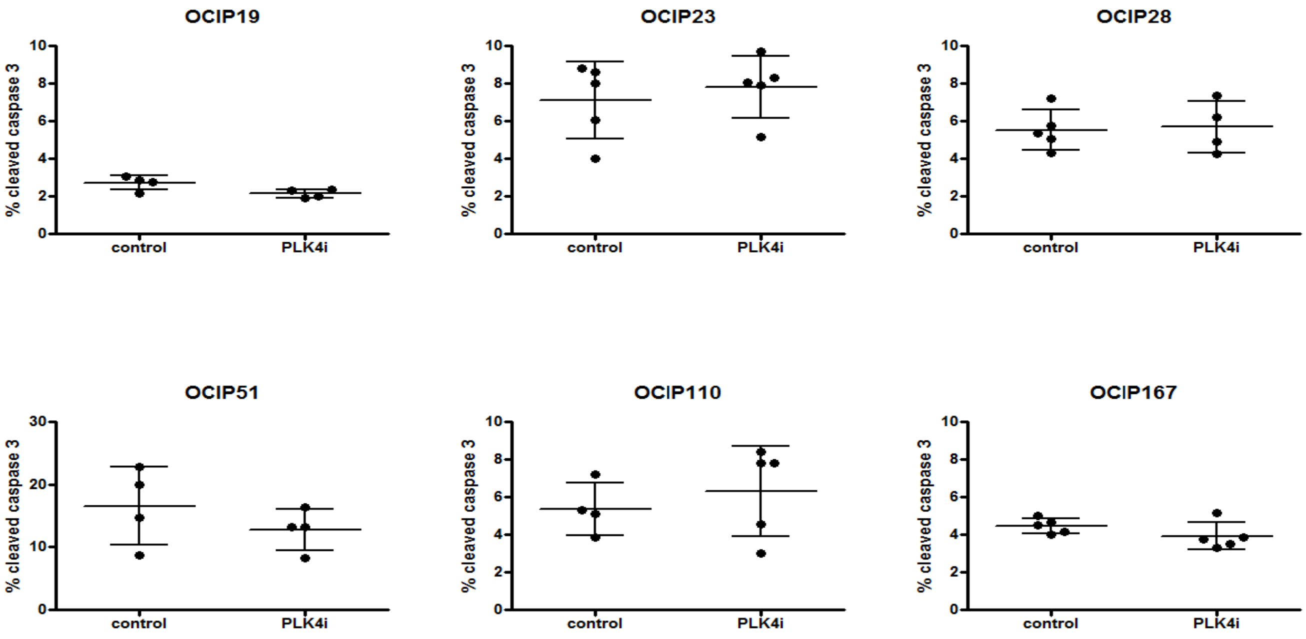

Supplementary Figure S2: Treatment with CFI-400945 results in growth arrest but not induction of apoptosis. Analysis of representative tumor sections stained for cleaved Caspase 3 (n=4-5).

Supplementary Table S1: Characterization of mitotic phases in response to treatment with the PLK-4 inhibitor CFI-400945

Model		Total pH3 positive nuclei/ mm^2		Prophase (%)		Metaphase (%)		Anaphase (%)		Telophase (%)	
		mean	SD	mean	SD	mean	SD	mean	SD	mean	SD
OCIP19	control	123.52	15.56	82.11	3.66	12.09	3.42	3.89	1.05	1.91	0.66
	PLK4i	72.69	9.43	86.75	3.15	8.02	2.57	3.48	0.83	1.75	0.84
OCIP23	control	385.85	70.00	73.46	1.92	17.93	2.73	5.2	0.62	3.42	0.35
	PLK4i	216.72	81.24	58.22	2.55	35.07	1.91	5.45	0.58	1.26	0.31
OCIP28	control	340.40	113.74	94.82	1.37	4.42	0.91	0.37	0.33	0.39	0.29
	PLK4i	115.47	88.17	96.51	0.70	3.1	0.76	0.08	0.03	0.31	0.06
OCIP51	control	381.16	181.57	78.57	1.77	16.93	1.94	3.21	0.08	1.32	0.11
	PLK4i	39.54	22.87	89.03	6.74	6.30	5.46	3.34	1.91	0	0
OCIP110	control	203.53	87.77	92.61	2.81	6.18	2.13	0.64	0.25	0.58	0.51
	PLK4i	125.82	66.84	96.72	1.96	3.22	2	0.07	0.13	0	0
OCIP167	control	232.75	87.10	95.17	1.3	3.48	0.86	0.34	0.24	1.01	0.47
	PLK4i	82.96	5.99	95.69	1.3	3.92	1.25	0.18	0.06	0.21	0.24

Supplementary Table S2: Statistical analysis of mitotic phases in response to treatment with the PLK-4 inhibitor CFI-400945

Model	Total pH3 positive nuclei/mm^2	Prophase	Metaphase	Anaphase	Telophase
OCIP19	p=0.029	p=0.2	p=0.11	p=0.69	p=0.89
OCIP23	p=0.057	p=0.029	p=0.029	p=0.69	p=0.029
OCIP28	p=0.057	p=0.11	p=0.11	p=0.041	p=1
OCIP51	p=0.029	p=0.029	p=0.029	p=1	p=0.77
OCIP110	p=0.34	p=0.11	p=0.11	p=0.026	p=0.02
OCIP167	p=0.029	p=0.49	p=0.89	p=0.47	p=0.059
